# Structure and photovoltaic properties of ZnO nanowire for dye-sensitized solar cells

**DOI:** 10.1186/1556-276X-7-260

**Published:** 2012-05-18

**Authors:** Ming-Cheng Kao, Hone-Zern Chen, San-Lin Young, Chen-Cheng Lin, Chung-Yuan Kung

**Affiliations:** 1 Department of Electronic Engineering, Hsiuping University of Science and Technology, Taichung, 412, Taiwan; 2 Department of Electrical Engineering, National Chung Hsing University, Taichung, 402, Taiwan

**Keywords:** ZnO, Nanowires, DSSC, Open-circuit voltage

## Abstract

Aligned ZnO nanowires with different lengths (1 to approximately 4 μm) have been deposited on indium titanium oxide-coated glass substrates by using the solution phase deposition method for application as a work electrode in dye-sensitized solar cells (DSSC). From the results, the increases in length of zinc oxide (ZnO) nanowires can increase adsorption of the N3 dye through ZnO nanowires to improve the short-circuit photocurrent (*J*_sc_) and open-circuit voltage (*V*_oc_), respectively. However, the *J*_sc_ and *V*_oc_ values of DSSC with ZnO nanowires length of 4.0 μm (4.8 mA/cm^2^ and 0.58 V) are smaller than those of DSSC with ZnO nanowires length of 3.0 μm (5.6 mA/cm^2^ and 0.62 V). It could be due to the increased length of ZnO nanowires also resulted in a decrease in the transmittance of ZnO nanowires thus reducing the incident light intensity on the N3 dye. Optimum power conversion efficiency (*η*) of 1.49% was obtained in a DSSC with the ZnO nanowires length of 3 μm.

## **Background**

Dye-sensitized solar cells (DSSCs) have been studied extensively as a potential alternative to conventional inorganic solid solar cells, by using nanocrystalline TiO_2_ sensitized with ruthenium polypyridine complexes or metal-free organic dyes as photoelectrodes [1-3]. Solar cells based on TiO_2_ nanoparticles with a size of 10 to 30 nm have been used as photoanodes with demonstrated 11% photovoltaic conversion efficiency [4]. Zinc oxide (ZnO) is another promising but less explored wide bandgap semiconductor oxide used for DSSC. It has similar energy levels to TiO_2_. More importantly, its much higher carrier mobility is more favorable for the collection of photo-induced electrons [5,6]. DSSCs based on TiO_2_ nanoparticles network usually consist of a porous layer which serves as the photoelectrode. The porous photoelectrode can provide large surface area for anchoring the light-capturing dye molecules to generate electron-hole pairs. However, in this kind of photoelectrode, the photogenerated electrons will interact with a lot of traps when they walk through the random network, which limits the efficiency of the DSSCs. Compared to TiO_2_, a ZnO semiconductor has a similar band gap energy and conduction band edge, making it a possible candidate for an effective nanorod-based DSSCs. ZnO nanowires as photoelectrodes show a higher conversion efficiency compared to those using the nanostructured films [7,8]. The single crystalline ZnO nanowires can significantly improve the electron transport in the photoelectrode by providing a direct conduction pathway for photogenerated electrons.

DSSCs based on ZnO nanowires have been prepared by many growth techniques, such as metal-organic chemical vapor deposition, hydrothermal method, vapor deposition and have been employed to grow ZnO nanowires [9-11]. In this paper, we report the controllable growth of ZnO nanowires through a simple and low-temperature solution phase deposition method. The effects of nanowire length on the material and optical properties of ZnO nanowires were investigated in details. Furthermore, ZnO nanowires with optimum material and electrical properties were successfully implemented in DSSCs to improve the photovoltaic performance.

## **Methods**

ZnO nanowires were fabricated by solution phase deposition method on the seeded indium titanium oxide (ITO) glass substrate. First, the ZnO thin films were deposited on ITO glass substrate using sol-gel methods. The ZnO thin films were then used as seed layers for growing nanowires. Zn(C_2_H_3_O_2_)_2_·2H_2_O and C_3_H_8_O_2_ were employed to synthesize the ZnO precursor. The concentration of ZnO was 0.5 M. Finally, the mixture was stirred at 60°C for 3 h in the water bath to form a transparent homogeneous mixture. After the 24 h aging at room temperature, the mixture keeps clear which can be utilized to deposited ZnO seed layer. The seed layer was deposited by spin coating onto the substrate with the rotation speed of 3,000 rpm for 30 s. Then, samples were performed by rapid thermal processing at 600°C for 2 min and ZnO seed layer was formed on the substrate. Thereafter, ZnO nanowires were grown by a 5 mM zinc acetate (Zn(C_2_H_3_O_2_)_2_·2H_2_O) solution mixed with hexamethylenetetramine (C_6_H_12_N_4_) at 90°C. The desired ZnO nanowire lengths of 1 to approximately 4 μm were achieved at growth time of 1, 2, 3 and 4 h, respectively. Finally, the samples were rinsed with deionized water and dried in air at room temperature to remove the solvent.

**Figure 1:**
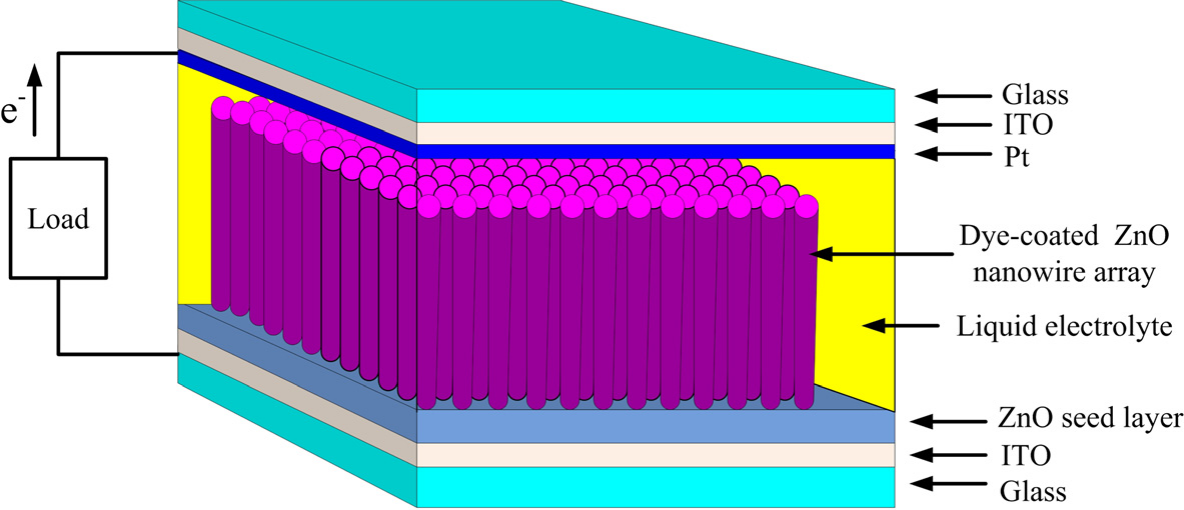
Schematic diagram of DSSC with ZnO nanowires work electrode.

The crystallization of ZnO nanowires was determined using Rigaku Dmax 2200 X-ray diffractometer with CuKα radiation (Rigaku Corporation, Tokyo, Japan) (λ = 1.5418 Å). The morphologies of the samples were characterized by field emission scanning electron microscopy (FE-SEM JEOL JSM-6700 F, JEOL Ltd., Tokyo, Japan). Photoluminescence spectroscopy was used to measure optical emissions from 350 to 600 nm using the He-Cd laser with wavelength of 325 nm. In order to sensitize ZnO nanowires, the ZnO electrodes were immersed in a 3 × 10^−4^ M solution of RuL_2_(NCS)_2_ (Solaronix, N3 dye, Rue de l’Ouriette, Aubonne, Switzerland) in ethanol for 2 h at room temperature. The electrolyte was composed of 0.5 M lithium iodide LiI/0.05 M iodine in acetonitrile. The device structure of DSSC is shown in Figure 1. The short-circuit photocurrent (*J*_sc_) and open-circuit voltage (*V*_oc_) were measured using a Keithley Model 2400 source measure unit (Keithley Instruments Inc., Cleveland, OH, USA) under an illumination of 100 mW/cm^2^ (Amplitude modulation 1.5 simulated light radiation) from a 1,000 W xenon lamp. Incident monochromatic photo-to-current conversion efficiency (IPCE) measurements were carried out using small band-pass filters to generate monochromatic light. The wavelength was controlled from 300 to 800 nm.

## **Results and discussion**

**Figure 2:**
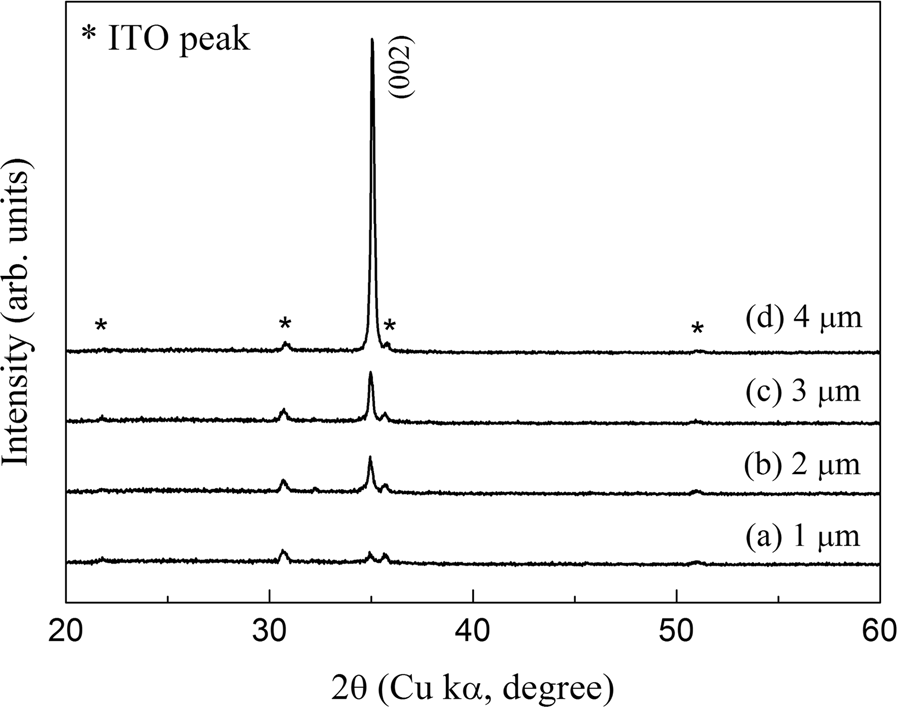
**X-ray diffraction pattern of ZnO nanowires with nanowire length.** Lengths are (**a**) 1 μm, (**b**) 2 μm, (**c**) 3 μm, and (**d**) 4 μm.

The crystallinity of grown ZnO nanowires on ITO substrate was investigated using X-ray diffraction. Figure 2 shows the two X-ray diffraction pattern (XRD) patterns of ZnO nanowires deposited on ITO-coated glass substrates with different lengths of 1 to approximately 4 μm, respectively. The peak in the XRD pattern reveals an oriented growth of ZnO nanowires in all the samples. We confirmed from the Joint Committee on Powder Diffraction Standard data (number 36-1451) that the diffraction from the planes corresponding to the 2 θ values was attributed to ZnO wurtzite structures. With respect to the crystallographic orientation, the most intense peak of ZnO corresponds to the (002) plane, indicating a strong preferential orientation along the [0001] direction. ZnO nanowires deposited for 4 h shows maximum diffraction intension to (002) plane and least to the others, highlight the significant growth of nanowires is oriented along *c*-axis. The increase of (002) peak intensity with growth time for ZnO nanowires indicates the enhancement of crystallization. The resulting observation can be inferred from scanning electron microscopy (SEM) observations. These results reveal that the nanowires, crystallized along the ZnO [0001] direction, were hexagonal prisms vertically aligned on the ITO substrate.

**Figure 3:**
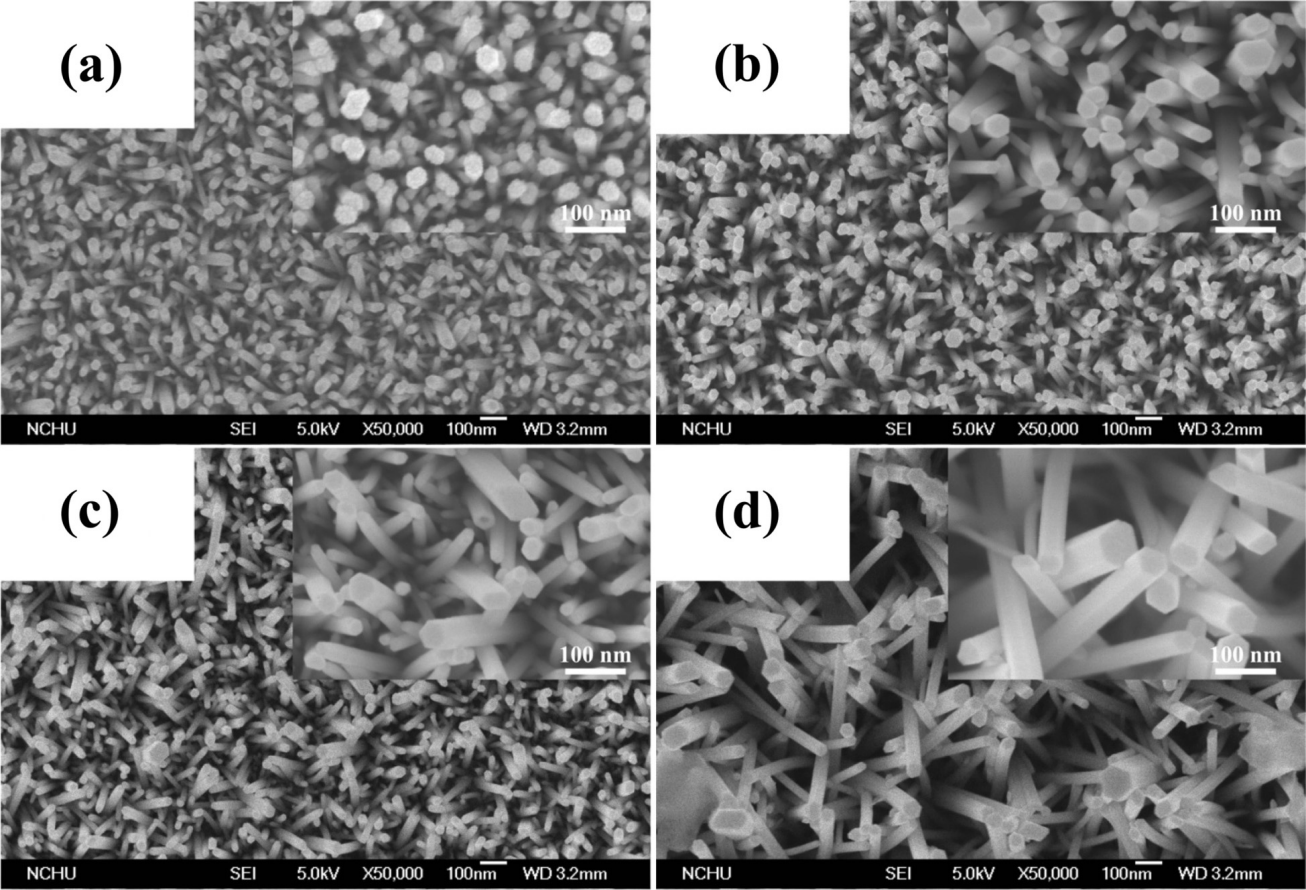
**SEM images of ZnO nanowires with nanowire length.** Lengths are (**a**) 1 μm, (**b**) 2 μm, (**c**) 3 μm and (**d**) 4 μm.

**Figure 4:**
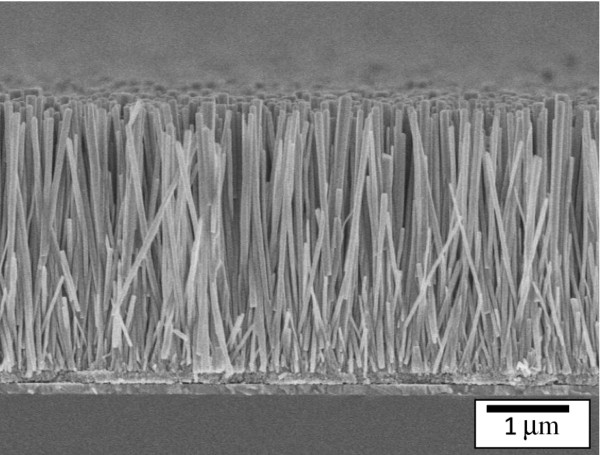
A cross-sectional photograph of ZnO nanowire with nanowire length of 3 μm.

Figure 3 shows the surface morphologies of ZnO nanowires with different lengths of 1 to approximately 4 μm. We found that the different growth time influenced the morphology and density of ZnO nanowires, leading to the different performance of the DSSCs based on ZnO nanowires. The top-view FE-SEM depicts images of samples prepared with 5 mM for 1, 2, 3, and 4 h on the ITO substrates with ZnO nanowires, as shown in Figure 3. In this study, ZnO seed layer and ZnO nanowires are all with wurtzite structure. Moreover, the ZnO seed layer is composed of circular nanoparticles. Therefore, ZnO nanowires have near cylindrical structure, which is shown in the inserted diagram in Figure 3. The diameter distributions between the ZnO nanowires for difference growth time have a significant difference which shows a growth of dense nanowires with tip diameter between 5 and 50 nm. The growth time is the key parameter to dominate the diameter of ZnO nanowires. By increasing the growth time, both the length and diameter of ZnO nanowires are increased. A cross-sectional SEM micrograph of the ZnO nanowire (3 μm) is shown in Figure 4. No evidence of a second-phase interfacial layer between the ZnO nanowires, ZnO seed layer, and the bottom ITO layer is found. The SEM micrograph also reveals that the nanowire is uniform, smooth, and crack-free on the surface. In addition, ZnO nanowire exhibits nanocrystalline and nanoporous structures which are composed of interconnected nanowires.

**Figure 5:**
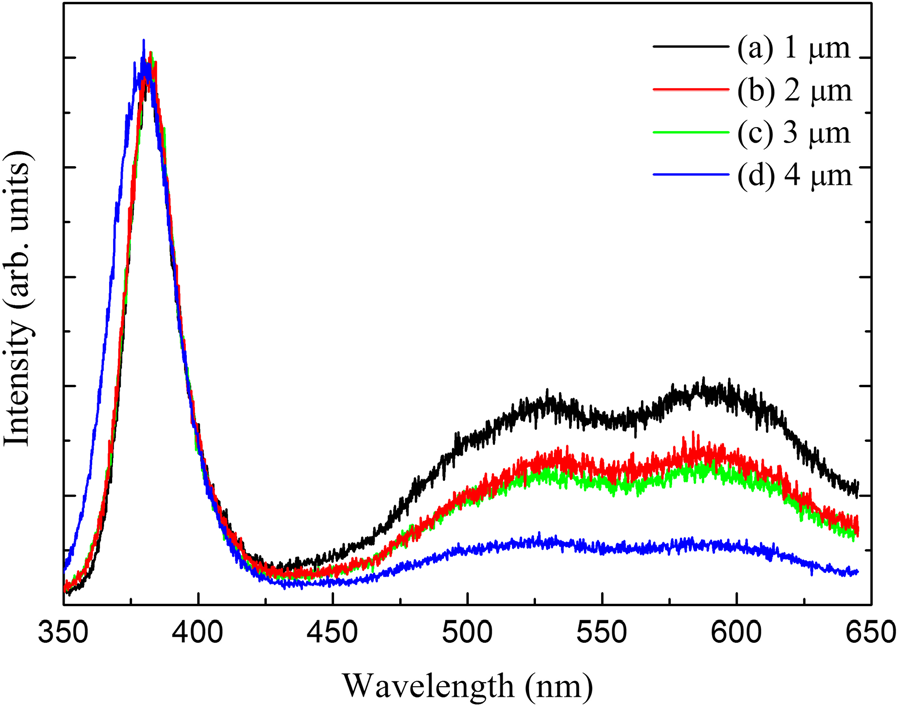
**Room temperature photoluminescence spectrum of ZnO nanowires with nanowire length.** Lengths are (**a**) 1 μm, (**b**) 2 μm, (**c**) 3 μm and (**d**) 4 μm.

Figure 5 shows the photoluminescence (PL) spectra of ZnO nanowires at room temperature. In the figure, the PL spectrum of ZnO nanowires has similar line-shape and consisted of two main parts: one is in the ultraviolet (UV) region, while the other is in the visible light region. The UV emission band centered at about 382 nm is originated from the exciton recombination corresponding to the near-band edge exciton emission of the wide bandgap ZnO, namely, the free excitons recombination through an exciton-exciton collision process [12,13]. The green-yellow emission is due to point defects such as oxygen vacancies or impurities which may be the results from the radial recombination of a photogenerated hole with an electron that belongs to a singly ionized oxygen vacancy [14,15]. The result can be explained by the change of the defect type as well as the decrease of the defect level related to growth time. Compared to the UV peak position of 4 μm nanowire in the PL spectrum, there is a red shift for the UV peak position of 1 μm nanowire. It is due to the defect density of 1 μm nanowire which is larger than 4 μm nanowire [16]. In addition, the green-yellow emission decreases with the increase of the length of ZnO nanowires which indicates the decrease of oxygen vacancies and Zn interstitial for the ZnO nanowires.

**Figure 6:**
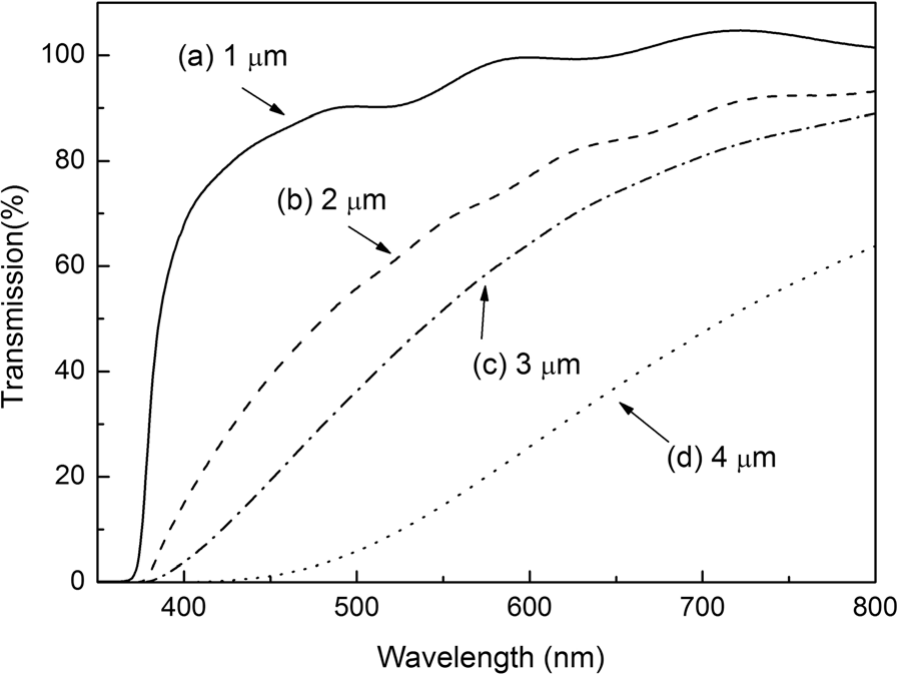
**Optical transmittance spectra of ZnO nanowires with nanowire length.** Lengths are (**a**) 1 μm, (**b**) 2 μm, (**c**) 3 μm and (**d**) 4 μm.

**Figure 7:**
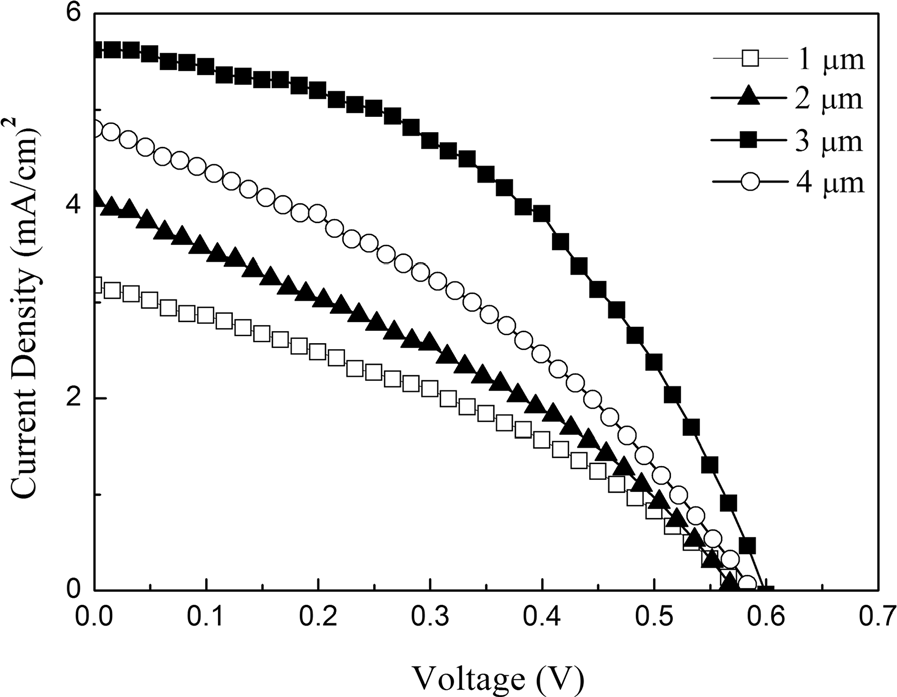
**Comparison of*J-V*curves of ZnO nanowires and photoelectrodes-based DSSCs.**

In this study, the transmittance of the glass/ITO/ZnO seed layer/ZnO nanowires samples (no N3 dye and electrolyte) was measured by using a UV-visible-infrared spectrometer. Figure 6 shows the optical transmittance spectra of ZnO nanowires with different length between 300 and 800 nm in wavelength. It can be seen that all nanowires have high transmittance, and the absorption edge is at about 300 nm. In addition, the transmittance of ZnO nanowires becomes lower as the nanowires actually become longer. The average transmittance of nanowires in Figure 6a,b,c,d at visible range about 90%, 80%, 70%, and 40%, respectively. Figure 7 shows the *I-V* curve for DSSC with different ZnO nanowires lengths (1.0 to approximately 4.0 μm). The *I-V* curve showed that the series resistance and shunt resistance of DSSC with ZnO nanowire length of 4 μm are decreased; this could explain the better blocking properties of the ZnO nanowire length of 3 μm. This also indicates that the density of ZnO nanowire length of 4 μm is less than that of 3 μm. Tables 1 also shows the various parameters of DSSC with different nanowire lengths. From these results, the *J*_sc_, *V*_oc_, and energy conversion efficiency *η* of DSSC increase with the increase of ZnO nanowire lengths from 1.0 to 3.0 μm. It is due to the larger nanowire lengths of ZnO nanowires, which result in higher adsorption of the N3 dye through ZnO/RuL_2_(NCS)_2_ layers. The enlarged surface area in the lengthened nanowires results in the increase of dye loading absorbed on ZnO nanowires. In addition, the multi-scattering effect between the nanowires enhances the optical path of the incident light in them. Both of these effects contribute to the improved energy conversion of solar cells with ZnO nanowire lengths from 1.0 to 3.0 μm. However, the *J*_sc_ and *V*_oc_ of DSSC with ZnO nanowires length of 4.0 μm (4.8 mA/cm^2^ and 0.58 V) are smaller than those of DSSC with ZnO nanowires length of 3.0 μm (5.6 mA/cm^2^ and 0.62 V), respectively. This can be explained by the lower transmittance of ZnO nanowires with the length of 4.0 μm to reduce the incident light intensity on the N3 dye (Figure 6). The lower incident light intensity on the ITO/ZnO nanowires/dye electrodes results into smaller amount of electrons by exciting dye molecules absorbed onto ZnO nanowires. The electrons injected from the dye molecules diffuse through the ZnO conduction band to the ITO will also decrease by decreasing the incident light intensity. The difference efficiency of ZnO nanowire lengths 1 to approximately 3 and 4 μm can be ascribed to a competition between dye loading absorbed and transmission effects. The optimum *η* of 1.49% with *J*_sc_ and *V*_oc_ of 5.6 mA/cm^2^ and 0.62 V, respectively, was obtained by ZnO nanowire with the length of 3.0 μm.

**Table 1:** Photovoltaic performances of DSSCs fabricated with various lengths of ZnO nanowires

Length of ZnO nanowires (μm)	*J*_sc_ (mA/cm^2^)	*V*_oc_ (V)	Fill factor (percent)	Efficiency (*η* percent)
1.0	3.2	0.56	35	0.62
2.0	4.1	0.57	36	0.84
3.0	5.6	0.62	43	1.49
4.0	4.8	0.58	38	1.06

In this study, the reliability of photocurrent generation concerned with ZnO nanowires-based DSSCs was measured through a chronoamperometry. The photocurrent-time characteristics were measured repeatedly at 60 s light source on and 60 s light source off in the range from 0 to 800 s. From the measurement results, within the scope measurement time of DSSC devices, stable photocurrent density of about 3.2, 4.0, 4.8, and 5.6 mA/cm^2^ can be obtained for ZnO nanowire lengths of 1, 2, 4, and 3 μm, respectively.

**Figure 8:**
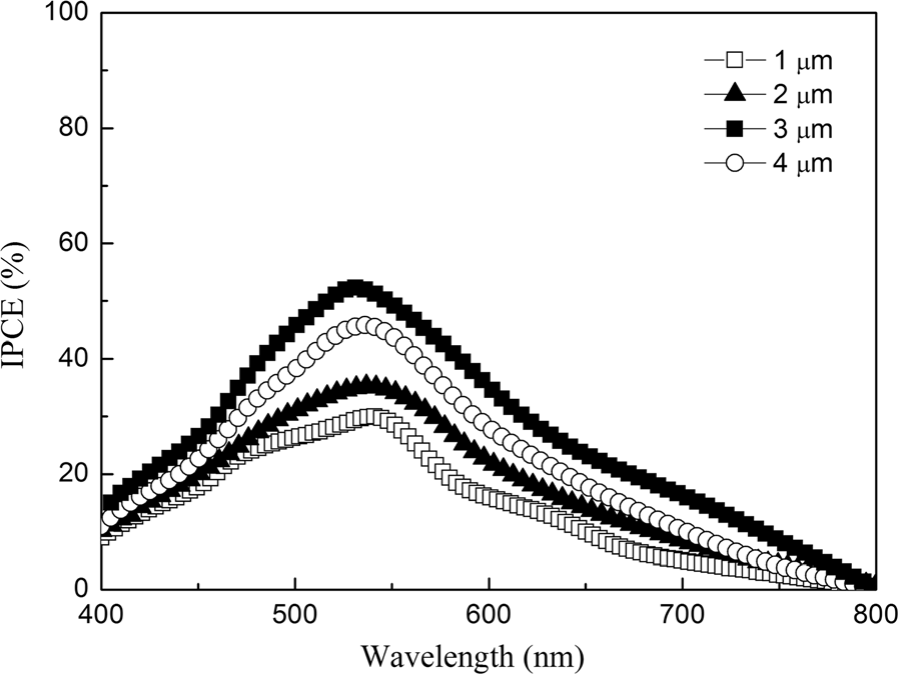
IPCE curve for DSSC with different ZnO nanowire lengths.

Figure 8 shows the IPCE of DSSC with different ZnO nanowire lengths as a function of wavelength. The IPCE is defined as the ratio of the number of electrons generated by light in the external circuit to the number of incident photons, as follows:

(1) $$\text{IPCE}=\frac{1,250\times \text{photocurrent density}(\mu {\text{A/cm}}^{2})}{\text{wavelength}(\text{nm})\times \text{photon flux}({\text{W/m}}^{2})}\text{,}$$

photocurrent density was determined at the short-circuit state. With the increased length of ZnO nanowires from 1.0 to 3.0 μm, the maximum IPCE values of DSSC increase from 30% up to 55% at 530 nm. However, the maximum IPCE value of DSSC with ZnO nanowires length of 3.0 μm (55%) was larger than that of DSSC with ZnO nanowires length of 4.0 μm (45%) at 530 nm. The higher IPCE value in the DSSC with ZnO nanowires length of 3.0 μm is attributed to the better transmittance of ZnO nanowires to increase the incident light intensity on the N3 dye.

## **Conclusions**

In this study, the influence of ZnO nanowires length on the performance of DSSCs was studied. With increasing the length of ZnO nanowires from 1.0 μm to 3.0 μm, the *J*_sc_ and *V*_oc_ values of DSSC increased from 3.2 to 5.6 mA/cm^2^ and from 0.56 to 0.62 V, respectively. However, the *J*_sc_ and *V*_oc_ of DSSC with ZnO nanowires length of 4.0 μm (4.8 mA/cm^2^ and 0.58 V) are smaller than those of DSSC with ZnO nanowires length of 3.0 μm (5.6 mA/cm^2^ and 0.62 V), respectively. This can be explained by the lower incident light intensity from the lower transmittance of ZnO nanowires with length of 4.0 μm. The corresponding results show that the obtained DSSC with ZnO nanowires length of 3.0 μm exhibited better photovoltaic properties.

## **Competing interests**

The authors declare that they have no competing interests.

## **Authors’ contributions**

MCK carried out the total study and wrote the manuscript. HZC was involved in the *I-V* curve and IPCE analyses of DSSC. SLY participated in the analyses of PL. CCL and CYK participated in the analyses of the XRD and FE-SEM. All authors read and approved the final manuscript.
